# A stratified random survey of the proportion of poor quality oral artesunate sold at medicine outlets in the Lao PDR – implications for therapeutic failure and drug resistance

**DOI:** 10.1186/1475-2875-8-172

**Published:** 2009-07-28

**Authors:** Sivong Sengaloundeth, Michael D Green, Facundo M Fernández, Ot Manolin, Khamlieng Phommavong, Vongsavanh Insixiengmay, Christina Y Hampton, Leonard Nyadong, Dallas C Mildenhall, Dana Hostetler, Lamphet Khounsaknalath, Latsamy Vongsack, Samlane Phompida, Viengxay Vanisaveth, Lamphone Syhakhang, Paul N Newton

**Affiliations:** 1 Food and Drug Department, Ministry of Health, Government of the Lao PDR, Vientiane, Lao PDR; 2 Division of Parasitic Diseases, US Centres for Disease Control and Prevention, Atlanta, USA; 3 School of Chemistry & Biochemistry, Georgia Institute of Technology, Atlanta, GA, USA; 4 Food and Drug Quality Control Centre, Ministry of Health, Government of the Lao PDR, Vientiane, Lao PDR; 5 GNS Science, Lower Hutt, New Zealand; 6 Centre for Malariology, Parasitology & Entomology, Government of the Lao PDR, Vientiane, Lao PDR; 7 Wellcome Trust – Mahosot Hospital – Oxford Tropical Medicine Research Collaboration, Microbiology Laboratory, Mahosot Hospital, Vientiane, Lao PDR; 8 Center for Clinical Vaccinology and Tropical Medicine, Churchill Hospital, University of Oxford, Oxford, UK

## Abstract

**Background:**

Counterfeit oral artesunate has been a major public health problem in mainland SE Asia, impeding malaria control. A countrywide stratified random survey was performed to determine the availability and quality of oral artesunate in pharmacies and outlets (shops selling medicines) in the Lao PDR (Laos).

**Methods:**

In 2003, 'mystery' shoppers were asked to buy artesunate tablets from 180 outlets in 12 of the 18 Lao provinces. Outlets were selected using stratified random sampling by investigators not involved in sampling. Samples were analysed for packaging characteristics, by the Fast Red Dye test, high-performance liquid chromatography (HPLC), mass spectrometry (MS), X-ray diffractometry and pollen analysis.

**Results:**

Of 180 outlets sampled, 25 (13.9%) sold oral artesunate. Outlets selling artesunate were more commonly found in the more malarious southern Laos. Of the 25 outlets, 22 (88%; 95%CI 68–97%) sold counterfeit artesunate, as defined by packaging and chemistry. No artesunate was detected in the counterfeits by any of the chemical analysis techniques and analysis of the packaging demonstrated seven different counterfeit types. There was complete agreement between the Fast Red dye test, HPLC and MS analysis. A wide variety of wrong active ingredients were found by MS. Of great concern, 4/27 (14.8%) fakes contained detectable amounts of artemisinin (0.26–115.7 mg/tablet).

**Conclusion:**

This random survey confirms results from previous convenience surveys that counterfeit artesunate is a severe public health problem. The presence of artemisinin in counterfeits may encourage malaria resistance to artemisinin derivatives. With increasing accessibility of artemisinin-derivative combination therapy (ACT) in Laos, the removal of artesunate monotherapy from pharmacies may be an effective intervention.

## Background

Artesunate is a key anti-malarial artemisinin derivative, developed in the People's Republic of China (China), vital for malaria treatment. It is widely used in South East (SE) Asia and increasingly in Africa for the treatment of *Plasmodium falciparum *malaria [1]. Although it should be used as a component of artemisinin-derivative based combination therapy (ACT), it is also widely and inappropriately sold as monotherapy [2,3]. There is good evidence that ACT, including either artesunate, artemether or dihydroartemisinin combined with a partner drug of relatively long biological half-life, is the most efficacious anti-malarial therapy, providing rapid parasite clearance and reduced gametocyte carriage and, in low or moderate transmission areas, reducing malaria incidence [1,4,5]. However, since the discovery of counterfeit artesunate in the late 1990s in mainland SE Asia [6,7], there has been concern that much of the artesunate used by patients is counterfeit, containing no or inadequate active ingredient [3,7-11]. In most countries the private sector is the major source of anti-malarial drugs and many patients have relied on artesunate monotherapy (2).

In the Lao People's Democratic Republic (Laos), 38% and 54% of oral artesunate collected, by convenience sampling, in 2000–2001 and 2002–2003, respectively, were counterfeit [7,9]. The prevalence in Laos reflects the situation in mainland SE Asia where 38% (2000–2001) and 53% (2002–2003) of oral artesunate were counterfeit [7,9]. In Asia, the artesunate tablets of one major producer, Guilin Pharmaceutical Co. Ltd. (Guilin, Guangxi autonomous region, China), has been targeted exclusively. The recent description of counterfeit artemisinin derivatives in six sub-Saharan African countries is of enormous public health concern [12-14]. Counterfeit artesunate containing sub-therapeutic quantities of artesunate has recently been described in Asia [3,15]. Such counterfeits, which may foil simple qualitative screening tests, will engender the selection and spread of artemisinin derivative resistant falciparum parasites, creating a disastrous situation for malaria control in Asia and, thereafter, in Africa. Sixteen different packaging types of counterfeit artesunate have been described with diverse chemical recipes including ineffective anti-malarials, such as chloroquine, antibiotics and potentially toxic pharmaceuticals [3,16,17]. A forensic analysis of the diversity of counterfeit artesunate suggested that at least some are produced in southern China, and that there are at least two factories or networks responsible. One putative network (the 'westerly' routes) seems to trade its 'products' through northern Burma into northern Laos and the Thailand/Burma border and another (the 'easterly' routes) through Vietnam into Cambodia and southern Laos [3]. Laos appears to be afflicted by 'products' flowing along both trade routes.

Public health research has neglected investigations of the quality of essential medicines, with few reliable data despite evidence suggesting that it is a major problem reducing the effectiveness of health care [18-21]. There are very few reliable published estimates of the prevalence of counterfeit, substandard or degraded medicines for any country [20-22]. Estimates of the prevalence of fake artesunate have all used 'convenience' sampling [7,9], which is potentially flawed by bias [23]. Biases may overestimate or underestimate the prevalence of poor quality drugs depending on whether the drug collectors, consciously or subconsciously, prefer to find or not find poor quality medicines. For quantitative estimates of the prevalence of counterfeit and substandard medicines and to allow comparisons through time, a standardized randomized sampling procedure, of sufficient sample size, is needed [23]. Differentiation between counterfeit, substandard or degraded products is important as this information is vital to allow medicine regulatory authorities (MRAs) to determine appropriate counter-measures. Only three published studies have apparently used random sampling [18,24-26].

Stratified random sampling of artesunate in Laos was therefore undertaken to estimate the countrywide prevalence of counterfeit or substandard medicine and to allow investigation of the impact of any subsequent interventions. This paper has two aims. First, to describe the methodology used to perform the random survey and second, to present data on the quality of oral artesunate in Laos.

## Methods

### Study locations and sampling

**Figure 1:**
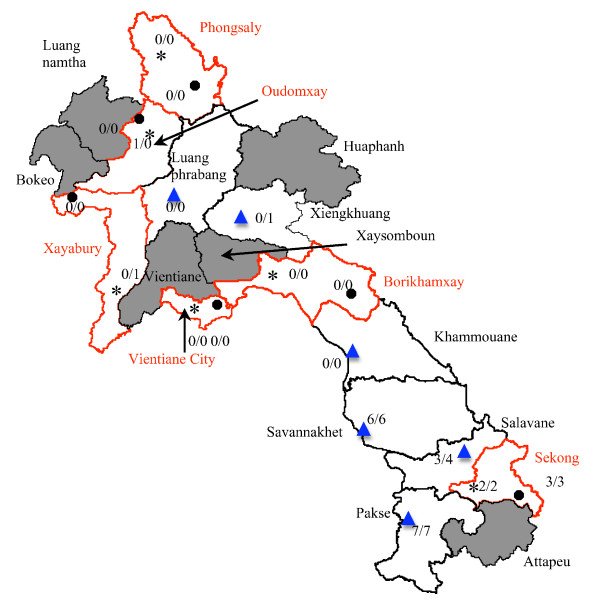
**Map of the Lao PDR showing distribution of collection sites with number of pharmacies selling counterfeit artesunate/number of pharmacies selling artesunate**. Provinces with red borders are those sampled in the first collection and stars indicate urban districts and solid circles indicate rural districts. Cities sampled in the second collection are shown as blue triangles. Provinces in grey were not sampled. Geographical names follow [44].

A stratified random sampling of medicines was performed to give an unbiased estimate of the proportion of outlets selling artesunate in Laos between March and June 2003. As it was not initially known how many licensed pharmacies and shops selling medicines there were in each district or province we did not perform a power calculation. Two sets of samples were collected. In the first collection, Laos was stratified into northern and southern provinces as falciparum malaria has a higher incidence in the south than in the north (see below, Figure 1) (the Vientiane Province/Vientiane City and Xiengkuang/Borikhamxay borders were the dividing line; in 2003 there were 18 provinces, now 17) and then by rural and urban districts. Provinces were selected first, with three of the 10 northern Lao provinces and three of the 8 southern Lao provinces randomly selected. The 42 districts in these selected provinces were classified as rural (27) and urban (15) on the basis of a consensus of the Lao Food and Drug Department (FDD) and Food and Drug Quality Control Centre (FDQCC) staff bearing in mind district population density, transport conditions and development. One urban and one rural district were randomly selected from each selected province. A statistician (Kasia Stepniewska), not involved in the field sampling, chose the locations using random number tables from lists of provinces and districts. The primary sampling units were the randomly chosen outlets in randomly chosen districts and provinces. Outlets were divided into licensed pharmacies and shops selling medicines. Itinerant drug sellers were not included. The study team met with the district and village authorities to enquire about licensed pharmacies and shops selling medicines within the study district and lists of outlets (Table 1, including district hospitals and clinics) in each study district were used for their random selection. As the sale of medicines from shops is illegal it is likely that they are underestimated but, as artesunate was not licensed in 2003, technically all artesunate sold was done so illegally.

**Table 1:** Frequency of genuine and fake artesunate at 180 pharmacies/shops selling medicines sample at random in Laos.

**First Collection – randomly selected licensed pharmacies and shops selling medicines in 12 districts in 6 Lao provinces**					
**Province **District	No. pharmacies/shops selling medicines recorded	No. pharmacies/shops selling medicines sampled	Pharmacies/shops from which artesunate bought	No (%) selling counterfeit artesunate	% slides positive for *P. falciparum *malaria/province in 2003 (number of slides checked)
**Northern Provinces**					
**Phongsaly** Phongsaly	8/3	7/3	0/0	-	1.5 (1,660)
**Phongsaly** May	2/3	2/3	0/0	-	
**Udomxay** Xay	24/2	7/2	1/0	1 (100)	12 (4,389)
**Udomxay** Namor	5/2	5/2	0/0	-	
**Xayabury** Paklai	24/3	7/3	1/0^a^	0 (0)	1.4 (21,486)
**Xayabuly **Khorb	7/2	7/2	0/0	-	
					
**Southern Provinces**					
**Vientiane City** Sisattanak	76/0	7/0	0/-	-	0.2 (1,008)
**Vientiane City** Maypaknum	15/0	7/0	0/-	-	
**Borikhamxay** Bolikanh	15/2	7/2	0/0	-	1.5 (274)
**Borikhamxay** Khamkueth	24/0	7/0	0/-	-	
**Sekong** Thateng	6/0	6/0	2/-	2 (100)	17.5 (7,149)
**Sekong** Dakcheung	4/0	4/0	3/-	3 (100)	
Total	210+17 = 227	73+17 = 90	7/0	6 (86)	5.9 (35,966)
**Second collection – randomly selected pharmacies in all 6 Lao cities (excluding Vientiane) of > 20,000 people**					
City	No. pharmacies recorded	No. pharmacies sampled	Pharmacies from which artesunate bought	No (%) selling counterfeit artesunate	% slides positive for *P. falciparum *malaria/province in 2003 (number of slides checked)
					
**Northern Provinces**					
Luangphrabang	51	15	0	-	4.6 (16,455)
Xiengkhuang	47	15	1^a^	0 (0)	0.4 (6,856)
**Southern Provinces**					
Thakhek	68	15	0/	-	2.5 (37,980)
Savannakhet	56	15	6	6 (100)^b^	11.8 (60,238)
Saravane	23	15	4^c^	3 (75)	11.8 (29,017)
Pakse	59	15	7^d^	7 (100)	6.4 (40,523)
Total	304	90	18	16 (89)	7.8 (191,069)
Overall Total	514+17 = 531	163+17 = 180	25	22 (88)	7.5 (227,035)

Geographical names follow [44]. For the First Collection the district in the first row is the urban district and the second row is the rural district.

^a ^genuine labeled as manufactured by Mekophar; ^b ^duplicates of artesunate bought at 5 pharmacies in Savannakhet (see Additional File 1);

^c ^genuine labeled as manufactured by Pharbaco ^d ^insufficient packaging available for one sample to judge whether counterfeit or genuine but chemistry demonstrated wrong active ingredients and therefore classed as counterfeit.

In each district, based on what was practical and without a power calculation, a sampling maximum of seven licensed pharmacies and three shops was planned. If there were less than these numbers of pharmacies/shops in a district, these were sampled without further arrangements. If there were more than seven licensed pharmacies or three shops selling medicines, those to be sampled were selected using a random number table. In order to direct the field teams to the randomly determined outlets, they were asked, once they had complete lists of outlets, to telephone the Microbiology Laboratory, Mahosot Hospital. The caller told the Laboratory the district name and the total number of pharmacies and shops in the lists. Using a random number table, seven licensed pharmacies and three shops selling medicines were selected and the caller given these two sets of numbers. A 'spare' number was also given for pharmacies and shops in case a pharmacy/shop was found closed after three attempts to sample.

A second collection was made as the first collection yielded few artesunate samples and we therefore chose to investigate larger population centers which would have more medicine outlets but with substantial rural populations and malaria. Fifteen randomly selected pharmacies in the central district of all six Lao cities (excluding Vientiane as this had already been sampled in the first collection) with a population >20,000 were sampled. The same telephone procedure as described above was used. Since drug regulation is more effective in cities, shops selling medicines were not expected to be present and therefore were not included.

For both collections, the drug sampling team consisted of two FDD/FDQCC Lao Government staff from Vientiane per province, working in cooperation with staff at the Provincial and District levels. The Vientiane staff was responsible for making up-to-date lists of pharmacies/shops according to the sampling plan and the actual drug sampling. The Provincial and District staff assisted with the updating of Government lists of pharmacies and creating lists of shops that sold medicines and in the drug collection through their local knowledge. Once the outlets to be sampled were identified by a telephone call to Vientiane, they were visited by the Lao 'mystery shopper', unaccompanied by anyone who might be linked to the drug regulatory authorities. The shoppers, of either gender, were dressed casually (representing mid-range of Lao socio-economic status) and stated in Lao language "*I would like to buy some medicine for my family – we are travelling – here is a list – may I see which ones you have so that I can choose?*". The list was handwritten in Lao on a scrap of paper. If only one make of a drug was offered the study customer asked "*Do you have any other makes, please?*". At least twenty tablets of oral artesunate were requested. If the outlet was closed, the mystery shopper visited a total of three times. If the outlet was closed at the third visit, the additional 'spare' code was used and that outlet visited. If the drug was given without a packet the pharmacist/shopkeeper was asked for the packet (except if the tablets were taken from jars this would not be requested).

The maximum number of makes or brands of artesunate available in each outlet was expected to be five. For outlets where more than one make of drug was available the shopper held a handwritten list containing a table for each shop giving the potential number of makes available (1–5), with a corresponding random number signifying which make to sample, counting the medicines from the left. If the drugs offered were a mixture of different makes or lot numbers or were past their expiry date they were still collected. The samples were stored at 4°C until analysis. The consensus among the investigators was that ethical review was not necessary.

### Sample analysis

#### Packaging

The physical appearance and printed text on packets, leaflet inserts and blisterpacks were examined and compared with known genuine artesunate. Packets, leaflets and blisterpacks were examined with an ×6 hand lens, ×100 stereomicroscope and with a handheld UV (375 nm) light source and then electronically scanned. Batch numbers, dates of expiry and manufacture, the colour, clarity and text of printing on the blisterpack, packet and leaflet (when present) were documented. Guilin Pharmaceutical Co. Ltd. kindly informed us which batch numbers were genuine. Samples were defined as counterfeit and substandard according to WHO definitions [27] and analysis of packaging was performed blinded to chemical results and vice-versa. Counterfeit samples were classified on the basis of the packaging details, especially the design of the fake hologram, into the different Types as described in [3].

#### Chemical and biological analysis

The Fast Red Dye test was performed according to Green *et al *[28,29]. Additionally, exposure of calcium carbonate to acetic acid in this test results in the release of carbon dioxide bubbles. Since Guilin brand tablets do not contain calcium carbonate (chalk), formation of bubbles would indicate a fake tablet composed of this chemical. High-performance liquid chromatography (HPLC) assays for artesunate were performed in 2003–2005 according to a previously described procedure [30]. For those samples found to contain artemisinin, chloroquine, pyrimethamine and sulphadoxine HPLC assays were performed according to the techniques described previously [30]. Mass spectrometry (MS) assays were performed in 2005 following a method previously reported [15]. The HPLC method used can detect >0.1 mg of active ingredients per tablet for the medicines investigated here. In contrast, MS can detect down to picogram-femtogram amounts of active ingredients per tablet. Artesunate reference standard (98–102% purity) used for the HPLC analysis was a generous donation from Mepha AG (Basel, Switzerland). As reported previously [3], four samples were analysed by X-ray diffractometry (XRD; X'Pert Pro X-ray diffractometer, Philips, Almelo, The Netherlands) and isotope ratio MS to determine the mineral composition and by microscopy for pollen and invertebrate remains.

## Results

180 outlets were sampled, representing 33.9% (180/531) of all pharmacies and shops selling medicines recorded in the study areas and 8.1% of all licensed pharmacies in Laos in 2003 (163/2,014) (Table 1). The majority of outlets sampled during the first collection were licensed pharmacies (81.5%). Shops selling artesunate were found in 7/12 (58.3%) districts. All pharmacies were Class 3 (i.e. 'with the licensee being neither pharmacist nor assistant pharmacist' [31].

Artesunate was found in 25 of all 180 outlets (13.9%). Considering only the pharmacies, 15.3% (25/163) sold artesunate. No artesunate was bought from shops selling medicines. Only one brand of artesunate was found in each pharmacy. Five pharmacies (20%) offered two blisterpacks of artesunate and the remaining 20 offered one. No 'spare' numbers were used as all selected outlets were open when visited.

### Packaging

All samples were sold in blisterpacks. By inspection of packaging, without knowledge of the chemical results, 26/30 (87% (95%CI 68–96%)) artesunate samples were counterfeit, three were genuine (10%) and the classification of one sample (12 Pas P2/1) could not be determined because key parts of the packaging were cut off before sale (Table 1, Figure 1).

The three genuine samples were labelled as made by Mekophar Chemical Pharmaceutical Joint-Stock Co. (Ho Chi Minh City, Vietnam) and Pharcabo (Hanoi, Vietnam) [32]. All the 26 counterfeits samples and the blisterpack of uncertain classification were labelled as made by Guilin Pharmaceutical Co. Ltd. For the 29 samples with an expiry date stated on the packaging (this was cut from one sample) the samples were bought before the expiry date. Of the 16 counterfeit artesunate types, Types 1 (one), 3 (one), 4 (three), 5 (seven), 7 (one), 8 (12) and 11 (one) [3] were represented. Of the five pharmacies that provided two blisterpacks, the packaging of the pairs was identical, except for one shop which sold both packaging Types 5 and 8. One counterfeit had a misspelling of Tablet as 'Tablte' on the blisterpack and one had an unusually long stated interval between manufacture and expiry date (nine years) (Additional file 1). Excluding Type 1, which lacking distinctive features is probably a mixture of different types, all the packaging types examined have only been described or have predominantly been described from the putative easterly distribution of fake artesunate in Laos, Cambodia and Vietnam [3].

### Chemical and botanical analysis

There was 100% agreement between the Fast Red dye test, HPLC, and MS for the 30 samples labelled as artesunate. All those classified as counterfeit, based on the packaging, contained no detectable artesunate. Chemical analysis of the sample with insufficient packaging (above) was shown by MS to contain pyrimethamine, sulphadoxine, and paracetamol, but no artesunate and was, therefore, counterfeit (Additional file 1). The combination of packaging and chemistry demonstrates that 27/30 (90%) (95%CI 72–97%) of artesunate samples were counterfeit. In terms of outlets, 22/25 (88%) (95% CI 68–97%) sold fake artesunate. The three genuine samples contained a median (range) artesunate content/tablet of 49.2 (45.1–50.3) mg (all had stated tablet content of 50 mg artesunate). The 27 counterfeit samples that underwent forensic MS were found to contain paracetamol (16), sulphadoxine (12), dimethylfumarate (6), metamizole (5), pyrimethamine (8), erythromycin (5), artemisinin (4), 2-mercaptobenzothiazole (3), chloramphenicol (2), chloroquine (1) and erucamide (1). The concentrations of artemisinin as determined by HPLC were 0.26, 4.50, 6.50 and 115.7 mg/tablet, chloroquine 14.7 mg/tablet, pyrimethamine 17.1, 16.0, 16.6 mg/tablet and sulphadoxine 409.6, 385.9, 413.9 mg/tablet (Additional file 1). Of 12 and 8 samples found by MS to contain sulphadoxine and pyrimethamine, respectively, sulphadoxine and pyrimethamine were found by HPLC in only three and three samples, respectively. The ratios of pyrimethamine to sulphadoxine, normally 1:20 in sulphadoxine-pyrimethamine (SP) coformulated tablets, were 1:24.0, 1:24.1 and 1:24.9 – suggesting that the counterfeits may have been formulated from powder after the co-drugs had been mixed to manufacture SP tablets. Of 4 samples examined with XRD, calcite was detected in three and starch in one. The stable isotope analysis of the calcite suggested a high temperature or volcanic origin as previously reported [3]. The results from the pollen analysis are consistent with a source of the fake artesunate in southern China, but do not prove this geographical origin as discussed in [3].

### Geographical distribution

**Figure 2:**
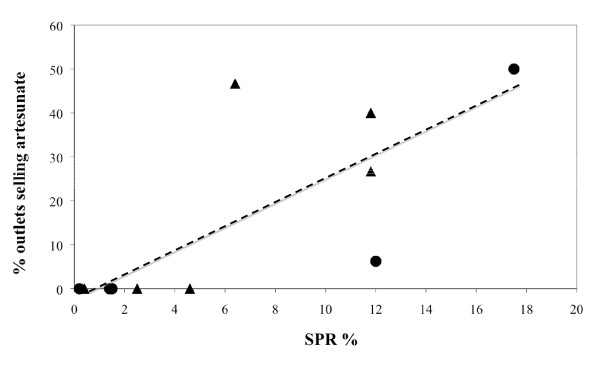
**Plot of slide positivity rate (SPR%) against the percentage of outlets selling artesunate for 12 Lao provinces**. R^2 ^= 0.60, P = 0.01. Circles indicate the first collection and triangles the second collection.

Artesunate was bought in seven of the 12 (58.3%) Lao provinces sampled, more frequently in southern Laos. The median (range) percentage of pharmacies selling artesunate in the sampled provinces were 0 (0–6.3) % and 27 (0–50) % in the northern and southern provinces, respectively (Figure 1). The seven sampled provinces with a slide positivity rate (all *Plasmodium *species) (SPR) <5% had a median (range) percentage of outlets selling artesunate of 0 (0–7)% and the five sampled provinces with an SPR of ≥5% had a median (range) percentage of outlets selling artesunate of 40 (6–50)%. With the exception of Udomxay, the more malarious sampled provinces were all in southern Laos – the median (range) SPR was 1.5 (0.4–12) % in the northern sampled provinces and 6.4 (0.2– 17.5) % in the southern sampled provinces. Provinces with more malaria (higher SPR) in 2003 were also those with a higher proportion of pharmacies selling artesunate (Table 1, Figure 2). In the first collection, the median (range) number of outlets per urban district was 22 (6–76) and 8 (4–17) per rural district. 3/6 urban and 1/6 rural districts contained outlets that sold artesunate and 3/4 and 3/3 of outlets in urban and rural districts, respectively, sold counterfeit artesunate.

## Discussion

Malaria remains an important clinical problem in Laos, with 14,997 patients with *P. falciparum *recorded in 2008 and five Lao provinces (all in the south) having >1,000 cases/year (unpublished data Centre for Malariology, Parasitology and Entomology, Vientiane (CMPE)). The random sampling survey reported here estimates that 13.9% of outlets in Laos sold oral artesunate but that it was more available in the malarious southern provinces. In an anti-malarial drug use survey in seven districts in the malarious provinces of Luangnamtha, Savannakhet and Attapeu Provinces in 2004 (unpublished data CMPE 2004), 5.5% of private sector and 11.1% of public sector outlets had artesunate tablets in stock. Both these surveys were conducted before the introduction of ACT by the Lao Government in 2005.

However, 88% of 25 pharmacies sampled in Laos in 2003 sold counterfeit artesunate. Substandard artesunate has been described from Thailand and Cambodia [3,33] but in Laos all the poor quality artesunate were counterfeit. This compares with previous estimates of the frequency of counterfeit artesunate of 38% in 2000–2001 [7] and 54% in 2002–2003 [9], but these studies used convenience and not random sampling. The results from convenience and random sampling are not comparable as different sampling frames and sampling methods were used. The random sampling methodology, which included 8% of licensed pharmacies in Laos, reduced the risk of bias – assuming the collectors followed the randomisation instructions – by eliminating the collectors' ability to decide which outlets to sample. That no artesunate was bought from shops selling medicines may not reflect the reality for patients and their relatives as the shop owners may have been more reluctant to take the risk of selling this product to an outsider.

Lot quality assurance sampling (LQAS) was recently proposed as the first step in assessing drug quality [23], but, as convenience sampling already suggested that there was a severe problem of counterfeit artesunate in Laos, a conventional randomized survey was performed. The majority of the details listed in the Medicine Quality Assessment Reporting Guidelines (MEDQUARG) [23] are reported. Limitations of this study include that we did not sample itinerant sellers who may also stock oral artesunate, the number of shops selling medicines sampled was relatively small and may have been underestimated, that we did not perform dissolution tests and urban and rural districts were not formally defined. Apart from a larger number of outlets in urban than rural districts there were no clear differences between the two district types. Although, a formal definition of urban districts was not used, there is no agreed definition of 'urban' [34] and all the urban districts sampled included rural farming areas. Of note the median (range) population of the six cities in the second collection was only 65,592 (20,239–200,462). Figure 2 includes both 'cities' and the urban and rural districts – they both demonstrate the same trend with increased availability of artesunate at higher provincial SPR. Lao cities and urban districts have small population size and are close to rural and malarious Laos, making the differences that one would expect in countries with larger disparities between urban and rural less pronounced in terms of medicine availability. Final chemical analysis was performed after the expiry date as stated on the packaging, although samples were stored at 4°C until analysis. MS did not give any strong evidence of artesunate degradation products, which would have been expected if this delay was significant.

Unsurprisingly, both genuine and counterfeit artesunate were both more commonly bought in the more malarious parts of Laos. Concentration of the survey in the more malarious southern areas would have increased power to find fake artesunate but in 2003 'clinical' malaria (ie without slide confirmation) was a diagnosis widespread in Laos. The different results obtained by MS and HPLC for the frequency of fakes containing sulphadoxine and pyrimethamine wrong active ingredients presumably reflect the lower limit of detection for these compounds by MS in comparison to HPLC. The findings are consistent with an origin of the Lao samples in the putative easterly fake artesunate trade network as suggested by the larger sample in [3]. If this is correct the recent disruption of the putative westerly trade network in SW China and northern Burma (Myanmar) [3] is unlikely to have reduced the frequency of fake artesunate in southern Laos.

Fake and substandard drugs are important but poorly recognized causes of death and disability to individual patients. They are also important to the whole of society as the exposure of pathogens, such as malaria parasites, to sub-therapeutic doses of antimicrobials will encourage the selection and spread of parasites resistant to the drugs [35]. The most resistant *P. falciparum *occur in SE Asia and multi-drug resistance to malaria remains a serious clinical problem. There is evidence that malaria parasites bearing high-level pyrimethamine resistance originally arrived in Africa from SE Asia [36].

Falciparum malaria resistant to artemisinin derivatives has started to appear on the Thailand/Cambodia border and urgent investigations and containment are being performed [37,38] (White NJ pers. comm.). Of considerable public health concern is that 14.8% of the fake artesunate collected contained artemisinin, from which artesunate is derived. The conventional dose of artemisinin in genuine tablets is 250 mg, whereas the artesunate fakes contained 0.26 – 115.7 mg artemisinin/tablet, suggesting that these fakes would be subtherapeutic and would engender artemisinin resistance. Counterfeit or substandard artesunate containing subtherapeutic quantities of artesunate have been found on the Thailand/Burma border [3,15], in Cambodia [3] and Thailand [33]. The mechanisms of resistance to artemisinin and the artemisinin-derivatives remain unclear but evidence for other anti-infective drugs suggests strongly that inadequate dosing is a major factor in selecting resistance. If poor quality medicines containing sub-therapeutic amounts of artesunate and/or artemisinin are widespread it should be assumed that they will increase the risk of the catastrophic spread of artesunate resistant malaria in Asia and thence to Africa.

It has been suggested that with the widespread use of ACT and withdrawal of ineffective medicines, such as chloroquine, malaria sensitivity to these drugs may return – as has been demonstrated in Malawi [39]. However, this will be dependent on the actual withdrawal of the abandoned medicines and with 44%, 30% and 4% of fake artesunate containing sulphadoxine, pyrimethamine and chloroquine, respectively, parasites are still being covertly exposed to these medicines. Conventional genuine chloroquine tablets contain 250 mg salt/tablet and SP contains pyrimethamine 25 mg and sulphadoxine 500 mg/tablet. As fake artesunate containing 14.7 mg chloroquine/tablet, and a median of 16.6 mg pyrimethamine and 403.1 mg/tablets sulphadoxine were found, it would be expected, depending on their pharmacokinetic/pharmacodynamic interactions, that these would select for the preferential survival of parasites with resistance mutations [35]. The wide range of non-standard concentrations of wrong active ingredients in tablets also suggests that these fake artesunate tablets are not recycled chloroquine, artemisinin and SP tablets but made of mixtures of powders by factories formulating anti-malarial drugs. That the ratios of pyrimethamine to sulphadoxine in the fake artesunate were close to the conventional ratio of SP tablets of 1:20 suggests that powder used in these fakes was being diverted from the manufacturer of SP. This raises the possibility that some of the fakes are being manufactured in a factory producing SP.

It was recently argued [40] that the relatively low prevalence of *P. falciparum *parasites in Laos with markers of antifolate resistance is a consequence of infrequent use of this class of anti-malarial in the country. This may change if current fake anti-malarials contain sulphadoxine and/or pyrimethamine. The presence of sulphadoxine in many of the counterfeits poses serious hazards for patients with allergies to sulpha drugs. Furthermore, 19% of fake artesunate contained metamizole, contraindicated in patients with glucose-6-phosphatase deficiency, which is common in Laos, and may precipitate haemolysis.

Monotherapy with artemisinin-derivatives is not recommended in most clinical situations [2,41] and ACT (artemether-lumefantrine) has been rolled out in Laos since 2005. However, recent evidence from adjacent Cambodia suggests that the majority of malaria patients still receive artesunate monotherapy despite subsidy of ACT in both the public and private sectors [2]. Comparable published data from Laos are not available. Investigations on the comparative availability of artesunate and ACT, and their quality, through the informal sector, such as grocery shops, and pharmacies in SE Asia may help guide appropriate interventions. There is an urgent need to determine the spectrum of unexpected active ingredients, especially artemisinin, among fake artesunate sold on the Thailand/Cambodia border area, which is ~400 km from southern Laos, out of concern that these may help drive resistance to the artemisinin derivatives [42]. An effective strategy to improve the anti-malarial therapy that patients receive and reduce the exposure of parasites to sub-therapeutic amounts of artemisinin/artesunate in fake artesunate, may be the removal of artesunate monotherapy (both genuine and fake) from pharmacies as ACT becomes available to all potentially malarious patients through the public and private sectors [43].

## Competing interests

The authors declare that they have no competing interests.

## Authors' contributions

SS designed study, collected samples, revised manuscript (ms). MDG designed study, supervised and performed chemical analysis. FMF designed study, supervised and performed chemical analysis. OM designed study, collected samples, revised ms. KP designed study, performed chemical analysis, revised ms. VI designed study, performed chemical analysis, revised ms. CYH performed chemical analysis, revised ms. LN performed chemical analysis, revised ms. DCM performed pollen analysis, revised ms. DH performed chemical analysis. LK performed chemical analysis, revised ms. SP designed study, revised ms. VV designed study, revised ms. LV designed study, performed chemical analysis, revised ms. LS designed study, collected samples, revised ms. PNN designed study and wrote first draft. All authors read and approved the final manuscript.

## Supplementary Material

Additional file 1 **Details of samples of artesunate blisterpacks collected in Laos by stratified random sampling**. The table describes the packaging détails and chemistry data for all the samples collected. Click here for file
