# Supplementary material for: A stratified random survey of the proportion of poor quality oral artesunate sold at medicine outlets in the Lao PDR – implications for therapeutic failure and drug resistance
Source: Malar J. 2009 Jul 28;8:172. doi: 10.1186/1475-2875-8-172 (PMC2734859; doi:10.1186/1475-2875-8-172)
Supplement: Additional file 1 — Details of samples of artesunate blisterpacks collected in Laos by stratified random sampling. The table describes the packaging détails and chemistry data for all the samples collected. [file 1475-2875-8-172-S1.pdf]

Additional file 1. Details of samples of artesunate blisterpacks collected in Laos by stratified random sampling. Data from some of these samples were published in [3,29]. Geographical names follow [44]. In Dye Test – Y = yellow (artesunate detected), C = clear (artesunate not detected) and Cg = clear with gas bubbles (suggesting calcium carbonate). ND= Not detected

Code	District/ Province	Stated manufacturer	Batch number Manufacture & Expiry date	Dye test	Packaging [Hologram Type]	Chemical content HPLC mg/tablet	Chemical content (MS, XRD)	Notes/pollen analysis
3 Pak 1/1	Paklai, Xayabury	Mekophar	0041202<br>-----<br>12/05	Y	Genuine	Artesunate 49.2	Artesunate	
2 Xai P/1 2220	Xay, Oudomxay	Guilin	990308	C	[1]	Artesunate ND<br>Chloroquine 14.7	Paracetamol, chloroquine, 2-mercaptobenzothiazole	
6 Duk P1/1 2221	Dakcheung, Sekong	Guilin	010401<br>04/01<br>04/04	C	[5]	Artesunate ND	Paracetamol, starch, sucrose, calcite 20%, $\delta^{13}C$ -9.60,$\delta^{18}O$ +5.9 ‰	Charcoal, cellular plant material, no pollen or spores
Duk P2/1 2223	Dakcheung, Sekong	Guilin	010401<br>04/01<br>04/04	C	[5]	Artesunate ND	Paracetamol	
Duk P3/1 2226	Dakcheung, Sekong	Guilin	010401<br>04/01<br>04/04	Cg	[8]	Artesunate ND	Paracetamol, dimethylfumarate, metamizole, starch, talc. Calcite 82%, $\delta^{13}C$ -9.62, $\delta^{18}O$ + 7.9 ‰	Charcoal. Parenchymatous cells, fungal material and hairs. No pollen or spores
6 Tat P3/1 2225	Thateng, Sekong	Guilin	010901<br>09/01<br>09/04	C	[4], 'Tablte'	Artesunate ND	2-mercaptobenzothiazole	

6 Tat P7/1 2228	Thateng, Sekong	Guilin	010401 04/01 04/04	Cg	[5]	Artesunate ND	Paracetamol	
8 Pek P 14/1 2230	Pek, Xiengkhuang	Mekophar	0031101 12 04	Y	genuine	Artesunate 45.1, 49.7, 50.1	Artesunate	
9 Sar P1/1 2232	Saravane, Saravane	Guilin	010401 04/01 04/04	Cg	[11]	Artesunate ND Artemisinin 0.26 Sulphadoxine ND	Paracetamol, sulphadoxine, erythromycin, dimethylfumarate, artemisinin	
9 Sar P5/1 2233	Saravane, Saravane	Guilin	010401 04/01 04/04	Cg	[5]	Artesunate ND	Paracetamol, erythromycin	
9 Sar P7/1 2234	Saravane, Saravane	Guilin	010401 04/01 04/04	Cg	[5]	Artesunate ND	Paracetamol, metamizole, erythromycin, dimethylfumarate	
9 Sar P11/1 2235	Saravane, Saravane	Pharbaco, Hanoi	010402 0405	Y	genuine	Artesunate 48.8, 50.3, 46.8	Artesunate	
11 Kha P3/1 2240 -1	Khanthabuly, Savannakhet [a]	Guilin	010401 04/01 04/04	Cg	[5]	Artesunate ND Artemisinin 4.5	Dimethylfumarate, paracetamol, artemisinin	
Kha 11.1 P3/2 2240-2	Khanthabuly, Savannakhet [a]	Guilin	010401 04/01 04/04	Cg	[8]	Artesunate ND Artemisinin 6.5 Sulphadoxine ND	Sulphadoxine, chloramphenicol, paracetamol, artemisinin	

Kha 11.1 P4/1 2241-1	Khanthabuly, Savannakhet [b]	Guilin	010401 04/01 04/04	C	[8]	Artesunate ND Pyrimethamine 17.1 Sulphadoxine 409.6	Pyrimethamine, sulphadoxine, erythromycin	
Kha 11.1 P4/2 2241-2	Khanthabuly, Savannakhet [b]	Guilin	010401 04/01 04/04	C	[8]	Artesunate ND Pyrimethamine 16.0 sulphadoxine 385.9	Pyrimethamine, sulphadoxine	
11 Kha P5/1 2242-1	Khanthabuly, Savannakhet [c]	Guilin	010401 04/01 04/04	C	[8]	Artesunate ND	starch, talc. Calcite 80%, $\delta^{13}C$ -9.57, $\delta^{18}O$ 8.7 ‰	Charcoal. Parenchymatous cells, fungal hyphae, invertebrate remains and hairs. *Alsophila* spores and *Pinus* pollen
11 Kha P5/2 2242-2	Khanthabuly, Savannakhet [c]	Guilin	010401 04/01 04/04	C	[8]	Artesunate ND	Chloramphenicol, metamizole	
11 Kha P7/1 2243-1	Khanthabuly, Savannakhet [d]	Guilin	020305 03/02 03/05	C	[4]	Artesunate ND	Metamizole	
11 Kha P7/2 2243-2	Khanthabuly, Savannakhet [d]	Guilin	020305 03/02 03/05	C	[4]	Artesunate ND	Metamizole	
11 Kha P11/1 2244-1	Khanthabuly, Savannakhet [e]	Guilin	010401 04/01 04/04	Cg	[8]	Artesunate ND Pyrimethamine ND Sulphadoxine ND	Pyrimethamine, sulphadoxine, paracetamol	

11 Kha P11/2 2244-2	Khanthabuly, Savannakhet [e]	Guiin	010401 04/01 04/04	Cg	[8]	Artesunate ND Sulphadoxine ND pyrimethamine ND	Sulphadoxine, pyrimethamine, paracetamol	
11 Kha P15/1 2245	Khanthabuly, Savannakhet	Guilin	000801 08/00 08/09	C	[7]	Artesunate ND Artemisinin 115.7	Artemisinin	
12 Pas P2/1 2262	Pakse, Champassack	Guilin	Cut off	---	Not classifiable	0 [f] Sulphadoxine ND pyrimethamine ND	Pyrimethamine, sulphadoxine, paracetamol	
12 Pas P10/1 2263	Pakse, Champassack	Guilin	010401 04/01 04/04	C	[8]	Artesunate ND Sulphadoxine ND	Dimethylfumarate, paracetamol, sulphadoxine, erythromycin	
12 Pas P11/1 2264	Pakse, Champassack	Guilin	010401 04/01 04/04	Cg	[8]	Artesunate ND Pyrimethamine ND Sulphadoxine ND	Pyrimethamine, sulphadoxine	
12 Pas P28/1 2265	Pakse, Champassack	Guilin	010401 04/01 04/04	C	[8]	Artesunate ND Pyrimethamine 16.6 Sulphadoxine 413.9	Paracetamol, pyrimethamine, sulphadoxine	

12 Pas<br>P61/1<br>2266	Pakse,<br>Champassack	Guilin	010401<br>04/01<br>04/04	C	[5]	Artesunate ND<br>sulphadoxine ND	Paracetamol,<br>sulphadoxine	
12 Pas<br>P62/1<br>2267	Pakse,<br>Champassack	Guilin	000902<br>09/00<br>09/03	C	[3]	0 [f]	Dimethylfumarate, 2-mercaptobenzothiazole and erucamide, Starch, talc	Numerous fine structureless organic particles, fungal hyphae and charcoal. A 5 mm long woody strand. *Artemisia*, *Ulmus* and *Casuarina* or *Carya* pollen
12 Pas<br>P64/1<br>2268	Pakse,<br>Champassack	Guilin	010401<br>04/01<br>04/04	C	[8]	Artesunate ND<br>Pyrimethamine ND<br>Sulphadoxine ND	Pyrimethamine,<br>sulphadoxine	

[a,b,c,d,e] Pairs of samples – each of pair bought at same pharmacy; [f] absence of artesunate determined only by mass spectrometry
